# Reactor Design and Optimization of *α*-Amino Ester Hydrolase- Catalyzed Synthesis of Cephalexin

**DOI:** 10.3389/fbioe.2022.826357

**Published:** 2022-03-02

**Authors:** Colton E. Lagerman, Martha A. Grover, Ronald. W. Rousseau, Andreas S. Bommarius

**Affiliations:** School of Chemical and Biomolecular Engineering, Georgia Institute of Technology, Atlanta, GA, United States

**Keywords:** *α*-amino ester hydrolase, Xanthomonas campestris pv. campestris, *ß*-lactam antibiotics, reactor modelling, reactor optimization

## Abstract

Pharmaceutical production quality has recently been a focus for improvement through incorporation of end-to-end continuous processing. Enzymatic *ß*-lactam antibiotic synthesis has been one focus for continuous manufacturing, and α-amino ester hydrolases (AEHs) are currently being explored for use in the synthesis of cephalexin due to their high reactivity and selectivity. In this study, several reactors were simulated to determine how reactor type and configuration impacts reactant conversion, fractional yield toward cephalexin, and volumetric productivity for AEH-catalyzed cephalexin synthesis. The primary reactor configurations studied are single reactors including a continuous stirred-tank reactor (CSTR) and plug flow reactor (PFR) as well as two CSTRS and a CSTR + PFR in series. Substrate concentrations fed to the reactors as well as enzyme concentration in the reactor were varied. The presence of substrate inhibition was found to have a negative impact on all reactor configurations studied. No reactor configuration simultaneously allowed high substrate conversion, high fractional yield, and high productivity; however, a single PFR was found to enable the highest substrate conversion with higher fractional yields than all other reactor configurations, by minimizing substrate inhibition. Finally, to further demonstrate the impact of substrate inhibition, an AEH engineered to improve substrate inhibition was simulated and Pareto optimal fronts for a CSTR catalyzed with the current AEH were compared to Pareto fronts for the improved AEH. Overall, reduced substrate inhibition would allow for high substrate conversion, fractional yield, and productivity with only a single CSTR.

## 1 Introduction

End-to end continuous processing has recently become a target for improving production quality in the pharmaceutical industry. Continuous processing often has distinct advantages over batch processing in terms of improvement in overall drug quality, efficiency, and controllability (Mascia et al., 2013; Lee et al., 2015; Myerson et al., 2015). *ß*-lactam antibiotics are promising candidates for continuous production due to the high volume of their consumption worldwide and the development of single-step enzymatic synthesis routes (Kasche 1986; Hernandez-Justiz et al., 1999; Youshko and Svedas 2000; Wegman et al., 2001; Youshko et al., 2002a; Elander 2003; Kallenberg et al., 2005; Chandel et al., 2008; Srirangan et al., 2013; Thakuria and Lahon 2013). Specifically, consumption of cephalosporins grew 94% between 2000 and 2010 and global production of cephalexin currently exceeds approximately 4,000 tons each year (Laxminarayan 2014; Van Boeckel et al., 2014). Recent studies have focused specifically on developing a continuous operation in simulations of continuous reactor designs using both enzymatic reaction and crystallization kinetics (Valencia et al., 2012; Encarnación-Gómez et al., 2016; McDonald et al., 2017; McDonald et al., 2019a; McDonald et al., 2019b; McDonald et al., 2019c; Cuthbertson et al., 2019; Salami et al., 2020).

β-lactam antibiotics are typically synthesized enzymatically using penicillin G acylase (PGA) due to its high thermostability, efficiency, and selectivity toward antibiotic synthesis. α-amino ester hydrolases (AEHs, EC 3.1.1.43), another class of enzymes, are also capable of stereoselective synthesis of *ß*-lactam antibiotics and have recently been shown to be particularly useful for cephalexin synthesis (Takahashi et al., 1972; Takahashi et al. 1974; Takahashi et al. 1977; Kato et al., 1980; Nam et al., 1985; Ryu and Ryu 1988; Polderman-Tijmes et al., 2002). AEH has been studied far less than PGA, and a kinetic model describing AEH-catalyzed synthesis of cephalexin has only recently been established (Lagerman et al., 2021). While AEH can synthesize cephalexin at a much faster rate than PGA and has a lower optimum pH of activity beneficial for *ß*-lactam stability (Barends et al., 2003), AEH also suffers from low thermostability and strong substrate inhibition (Blum and Bommarius 2010; Blum et al., 2012; Lagerman et al., 2021). While PGA is currently the favored enzyme for *ß*-lactam antibiotic synthesis, the synthesis potential of AEH has not been fully realized. Overall, cephalexin is the strongest candidate for synthesis by AEH, and understanding how AEH can be used in common reactor configurations is a prerequisite for developing AEH-catalyzed synthesis processes.

Well-developed kinetic models for the enzyme(s) involved in a reactor are required for successful prediction and design of optimal reactor configuration prior to construction and operation. Useful kinetic models describe reaction kinetics across a wide range of conditions relevant to large-scale processes. While numerous studies for simulation of enzymatic reactors exist, many models are generalized or developed for single-substrate or single-product reactions (Vasic-Racki et al., 2003; Harmand and Dochain 2005; Andrić et al., 2010; Lindeque and Woodley 2019), whereas enzymatic synthesis of *ß*-lactam antibiotics is a complex reaction network with multiple substrates, products, and inhibitions (Youshko and Svedas 2000; Youshko et al., 2002b; McDonald et al., 2017; Lagerman et al., 2021). The complexities of *ß*-lactam synthesis by AEH render even the simplest reactor design studies both non-trivial and necessary prior to reactor construction.

Enzymatic reactor modelling also allows for development of whole process models and coupled reaction-isolation systems in addition to reactor optimization. Accurate models for downstream processing of active pharmaceutical ingredients (APIs) rely on proper modelling of all upstream units, including reactors. Several studies have discussed complex reaction-isolation systems (Salami et al., 2020; Ma et al., 2021) as well as upstream process modelling for generation of reaction substrates (Caşcaval et al., 2012) and downstream process modelling from fermentation broths (Likozar et al., 2012; Likozar et al., 2013), which could all be coupled with reactor modelling for whole process models of API production and isolation.

In this work, reactor design considerations were studied for continuous synthesis of cephalexin using previously determined kinetics for an AEH from *Xanthomonas campestris pv. campestris.* The effects of inlet substrate concentrations, reactor enzyme concentration, and reactor configuration including the use of reactors in series were the primary focus. Multiple combinations of continuous stirred-tank reactors (CSTRs) and plug flow reactors (PFRs) were compared in terms of reactant conversion, fractional yield toward cephalexin, and volumetric productivity. To demonstrate the current limitations of AEH and demonstrate how reactor engineering and improvements in catalytic properties through protein engineering can positively impact the system, several reactor configurations were studied. Several substrate and enzyme concentrations were considered. Finally, a CSTR operated with an engineered AEH demonstrating reduced substrate inhibition was simulated to demonstrate the synthetic potential of an improved AEH.

## 2 Methods

### 2.1 Model Development

The reaction model used in this study is based on the mechanism and kinetics for AEH-catalyzed cephalexin synthesis previously studied (Lagerman et al., 2021). Briefly, AEH catalyzes the synthesis of cephalexin by direct coupling of two substrates: an activated acyl donating electrophile, phenylglycine methyl ester (PGME), and a *ß*-lactam ring containing nucleophile, (7-ADCA). In addition to the primary synthesis reaction, AEH also catalyzes the hydrolysis of PGME into the byproduct phenylglycine (PG) as well as hydrolysis of cephalexin into PG and 7-ADCA. Substrate inhibition by PGME had been found to have a significant impact on the synthesis potential of AEH at high concentrations of PGME through both competitive inhibition to form a nonreactive species, *EPGME·PGME,* as well as a partial competitive inhibition that still allows for hydrolysis of a PGME-bound acyl-enzyme complex, *EAPGME,* to PG (Scheme 10).

**SCHEME 1:**
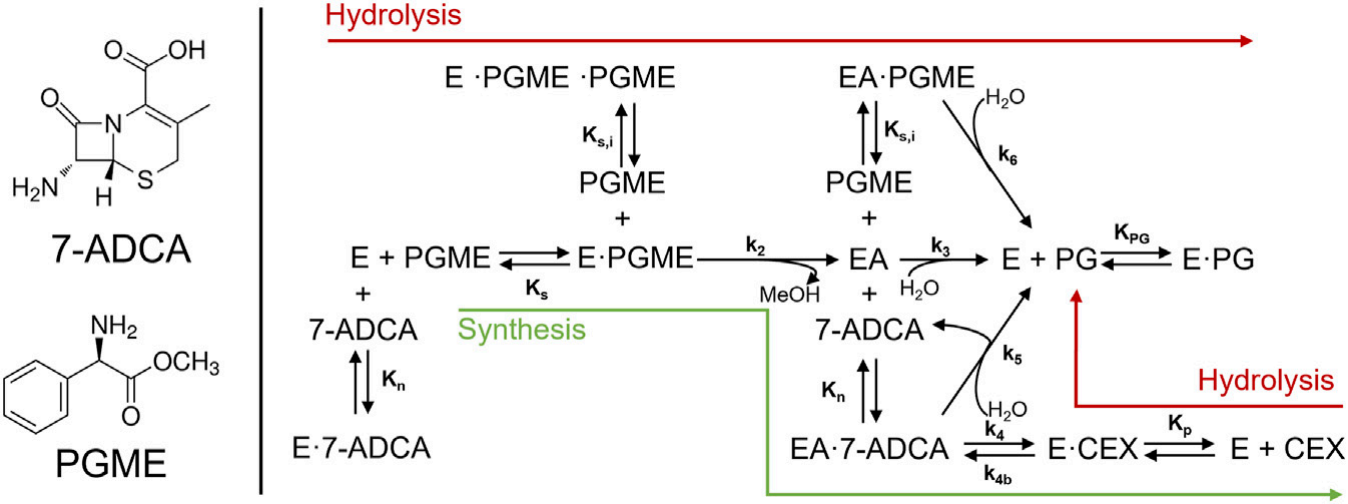
Reaction network from Lagerman *et al.* describing the reactions involved in the synthesis of cephalexin catalyzed by AEH. In addition to the primary synthesis reaction (green arrow), AEH catalyzes both the hydrolysis of PGME as well as cephalexin.

**FIGURE 1:**
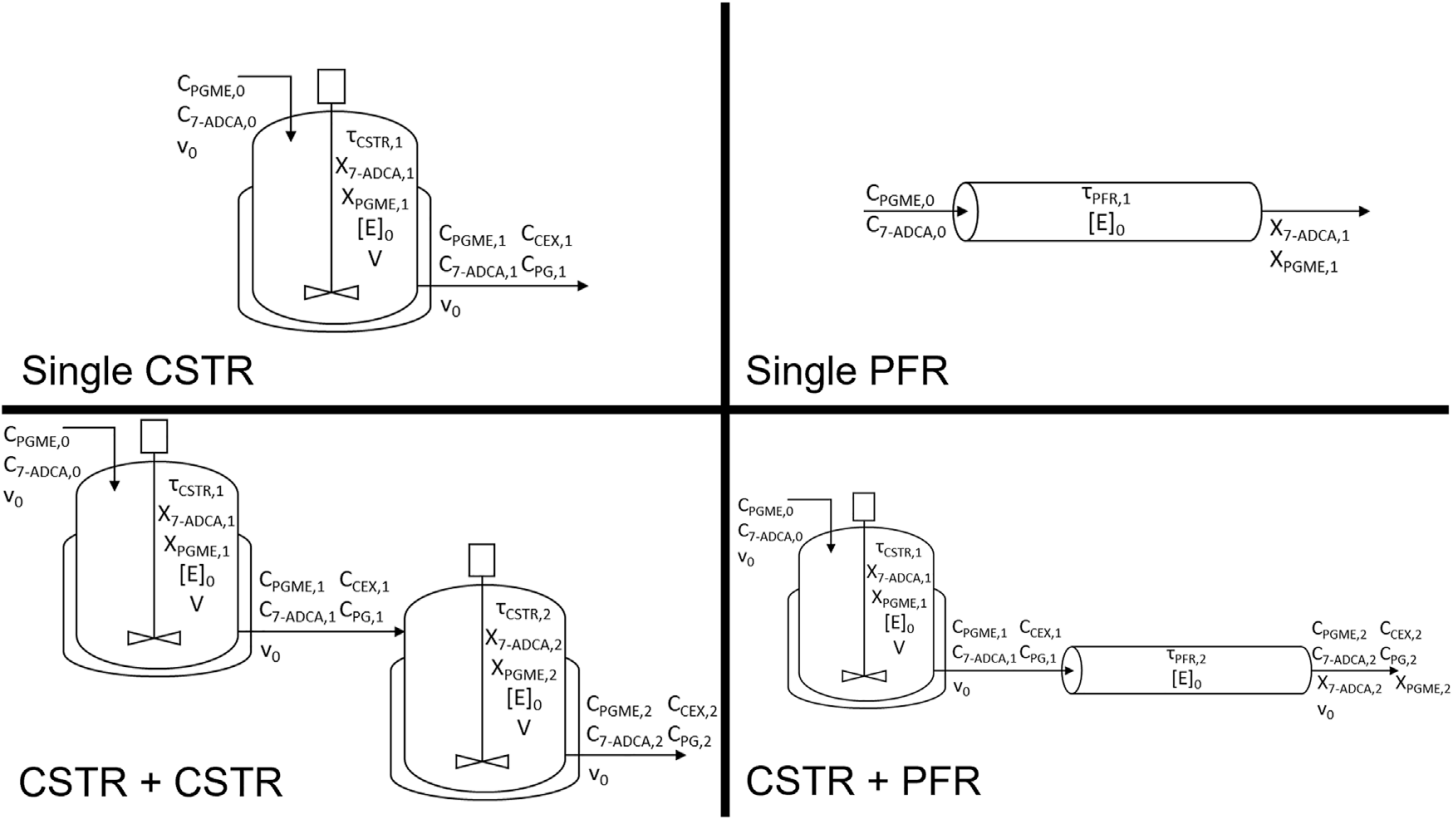
Reactor configurations simulated and corresponding variables tracked for each system. Reactor configurations include a single CSTR, single PFR, CSTR + CSTR, and a CSTR + PFR.

The rate equations for each reactant and product involved in the reaction were formulated as a system of differential equations connected through the acyl group and *ß*-lactam nuclei mass balances as previously described (Lagerman et al., 2021). Each reactor simulation was solved by setting the inlet concentrations of 7-ADCA (*C*_7-ADCA,0_) and PGME (*C*_PGME,0_) and the reactor enzyme concentration (*[E]*_*0*_). Reactor configurations were set by the respective design equations for each reactor system as described below. Residence time (τ), productivity (space-time yield, *s.t.y.*), and fractional yield were then solved and studied as the primary reactor metrics. Both a CSTR and a PFR were simulated using the rate equations from the model described above. In addition to the single reactors, two CSTRs in series as well as a CSTR followed by a PFR were also considered (Figure 1). All CSTRs were assumed to be well-mixed, implying that reactor and product concentrations in CSTR outlets are equal to their concentrations in the bulk reactor. The system of equations was solved using *vpasolve* in MATLAB R2021b for the single CSTR and two CSTR simulations. Systems containing a PFR were solved using *ode45* in MATLAB. All rate equations were calculated using previously determined model parameters (Lagerman et al., 2021).

Mass balances were derived for 7-ADCA and PGME consumption in a single CSTR and written in residence time format (Fogler 1999). To develop the design equation, each derived mass balance was set equal to the other, as a single CSTR configuration must satisfy both mass balances simultaneously. For the single CSTR (Scheme 10), the design equation

$$\begin{array}{c} \frac{X_{7-\mathrm{ADCA},1}C_{7-\mathrm{ADCA},0}}{\left(-R_{7-\mathrm{ADCA},1}\right)}=\frac{X_{\mathrm{PGME},1}C_{\mathrm{PGME},0}}{\left(-R_{\mathrm{PGME},1}\right)} \end{array}$$ (1)

equates the mass balance for 7-ADCA consumption (left-hand side of Eq. 1) and PGME consumption (right-hand side of Eq. 1) in a single CSTR. *X*_*PGME,1*_ is PGME conversion, *X*_*7-ADCA,1*_ is 7-ADCA conversion, and $R_{PGME,1}$ and $R_{7-ADCA,1}$ are the rate equations for PGME and 7-ADCA evaluated at the CSTR exit conditions such that

$$\begin{array}{c} R_{\mathrm{PGME},1}=-\frac{k_{2}\left[E\right]C_{PGME,1}}{K_{s}}\\ -\frac{k_{6}C_{PGME,1}}{K_{s,i}}\left(\frac{k_{2}\left[E\right]C_{PGME,1}}{K_{s}}+\frac{k_{4b}\left[E\right]C_{CEX,1}}{K_{p}}\right)\\ \left(\frac{1}{\frac{k_{4}C_{7-ADCA,1}}{K_{n}}+\frac{k_{5}C_{7-ADCA,1}}{K_{n}}+\frac{k_{6}C_{PGME,1}}{K_{si}}+k3}\right) \end{array}$$ (2)

$$\begin{array}{c} R_{7-\mathrm{ADCA},1}=-\frac{k_{2}\left[E\right]C_{PGME,1}}{K_{s}}\\ +\left(\frac{\left[E\right]}{\frac{k_{4}C_{7-ADCA,1}}{K_{n}}+\frac{k_{5}C_{7-ADCA,1}}{K_{n}}+\frac{k_{6}C_{PGME,1}}{K_{si}}+k3}\right)\\ \left(\frac{k_{2}C_{PGME,1}}{K_{s}}\;+\frac{k_{4b}C_{CEX,1}}{K_{p}}\right)\left(k_{3}+\frac{k_{5}C_{7-ADCA,1}}{K_{n}}\right) \end{array}$$ (3)

where [E] is the concentration of free AEH in the system derived from an enzyme balance (Supplementary Equation S3). Values for all kinetic parameters can be found in the Supplementary Table S1. It should be noted that all rate equations account for all reactions in the network shown in Scheme 10. For example, $R_{CEX}$ accounts for both cephalexin production and consumption and $R_{7-ADCA}$ accounts for both 7-ADCA production and consumption. Each rate is evaluated with the outlet concentrations of PGME, 7-ADCA, cephalexin, and PG ( $C_{PGME,1},C_{7-ADCA,1},C_{CEX,1},C_{PG,1}$ , respectively) and the concentration of AEH in the reactor, ${\left[E\right]}_{0}$ . The additional equations to describe the single CSTR include rate equations for cephalexin and PG ( $R_{CEX,1}$ and $R_{PG,1}$ ) and the outlet reactor concentrations of PGME, 7-ADCA, cephalexin, and PG ( $C_{PGME,1},C_{7-ADCA,1},C_{CEX,1},C_{PG,1}$ , respectively) were solved simultaneously with the design equation such that

$$\begin{array}{c} R_{\mathrm{CEX},1}=-R_{7-\mathrm{ADCA},1} \end{array}$$ (4)

$$\begin{array}{c} R_{\mathrm{PG},1}=-R_{\mathrm{PGME},1}-R_{\mathrm{CEX},1} \end{array}$$ (5)

$$\begin{array}{c} C_{\mathrm{PGME},1}=C_{\mathrm{PGME},0}-C_{\mathrm{PGME},0}X_{\mathrm{PGME},1} \end{array}$$ (6)

$$\begin{array}{c} C_{7-\mathrm{ADCA},1}=C_{7-\mathrm{ADCA},0}-C_{7-\mathrm{ADCA},0}X_{7-\mathrm{ADCA},1} \end{array}$$ (7)

$$\begin{array}{c} C_{\mathrm{CEX},1}=X_{7-\mathrm{ADCA},1}C_{7-\mathrm{ADCA},0} \end{array}$$ (8)

$$\begin{array}{c} C_{\mathrm{PG},1}=X_{\mathrm{PGME},1}C_{\mathrm{PGME},0}-C_{\mathrm{CEX},1} \end{array}$$ (9)

Finally, residence time ( $\tau_{CSTR}$ ), fractional yield, and productivity were calculated as

$$\begin{array}{c} \tau_{\mathrm{CSTR},1}=\frac{X_{7-\mathrm{ADCA},1}C_{7-\mathrm{ADCA},0}}{\left(-R_{7-\mathrm{ADCA},1}\right)} \end{array}$$ (10)

$$\begin{array}{c} \mathrm{Fractional Yield}=\frac{C_{\mathrm{CEX},1}}{C_{\mathrm{PGME},0}-C_{\mathrm{PGME},1}} \end{array}$$ (11)

$$\begin{array}{c} \mathrm{Productivity}\;\left(g/L/\mathrm{hr}\right)=\frac{\left(347.4\frac{g}{\mathrm{mol}}\right)\left(60\frac{\min}{\mathrm{hr}}\right)C_{\mathrm{CEX},1}}{\left(1000\frac{\mathrm{mmol}}{\mathrm{mol}}\right)\tau_{\mathrm{CSTR},1}} \end{array}$$ (12)

The PFR was simulated by solving the rate equations simultaneously in MATLAB using *ode45* to solve the system of rate equations for each substrate and product and obtain the outlet concentrations of all species at a wide range of residence times corresponding to the full conversion profiles of both substrates. At each residence time ( $\tau_{PFR,1}$ ), 7-ADCA and PGME conversion were calculated as

$$\begin{array}{c} X_{7-\mathrm{ADCA},1}=\frac{C_{7-\mathrm{ADCA},0}-C_{7-\mathrm{ADCA},1}}{C_{7-\mathrm{ADCA},0}} \end{array}$$ (13)

$$\begin{array}{c} X_{\mathrm{PGME},1}=\frac{C_{\mathrm{PGME},0}-C_{\mathrm{PGME},1}}{C_{\mathrm{PGME},0}} \end{array}$$ (14)

where $C_{7-ADCA,1}$ and $C_{PGME,1}$ are the outlet 7-ADCA and PGME concentrations for a given $\tau_{PFR,1}$ . Finally, fractional yield was calculated using Eq. 11 as for a CSTR and productivity was calculated as

$$\begin{array}{c} \mathrm{Productivity}\;\left(g/L/\mathrm{hr}\right)=\frac{\left(347.4\frac{g}{\mathrm{mol}}\right)\left(60\frac{\min}{\mathrm{hr}}\right)C_{\mathrm{CEX},1}}{\left(1000\frac{\mathrm{mmol}}{\mathrm{mol}}\right)\tau_{\mathrm{PFR},1}} \end{array}$$ (15)

For the two-CSTR system, the design equation for the first CSTR remains the same (Eq. 1) and the second CSTR design equation derived from a mass balance around the second CSTR is

$$\begin{array}{c} \frac{\left(X_{7-\mathrm{ADCA},2}-X_{7-\mathrm{ADCA},1}\right)C_{7-\mathrm{ADCA},0}}{\left(-R_{7-\mathrm{ADCA},2}\right)}=\frac{\left(X_{\mathrm{PGME},2}-X_{\mathrm{PGME},1}\right)C_{\mathrm{PGME},0}}{\left(-R_{\mathrm{PGME},2}\right)} \end{array}$$ (16)

where $X_{7-ADCA,2}$ and $X_{PGME,2}$ are the total 7-ADCA and PGME conversions after leaving the second CSTR and $R_{7-ADCA,2}$ and $R_{PGME,2}$ are the rates of consumption of 7-ADCA and PGME. To solve the CSTR + CSTR system, Eqs. 1–9) to describe the first CSTR as well as Eq 16 and

$$\begin{array}{c} R_{\mathrm{PGME},2}=-\frac{k_{2}\left[E\right]C_{PGME,2}}{K_{s}}-\frac{k_{6}C_{PGME,2}}{K_{s,i}}\\ \left(\frac{k_{2}\left[E\right]C_{PGME,2}}{K_{s}}+\frac{k_{4b}\left[E\right]C_{CEX,2}}{K_{p}}\right)\\ \left(\frac{1}{\frac{k_{4}C_{7-ADCA,2}}{K_{n}}+\frac{k_{5}C_{7-ADCA,2}}{K_{n}}+\frac{k_{6}C_{PGME,2}}{K_{si}}+k3}\right) \end{array}$$ (17)

$$\begin{array}{c} R_{7-\mathrm{ADCA},2}=-\frac{k_{2}\left[E\right]C_{PGME,2}}{K_{s}}\\ +\left(\frac{\left[E\right]}{\frac{k_{4}C_{7-ADCA,2}}{K_{n}}+\frac{k_{5}C_{7-ADCA,2}}{K_{n}}+\frac{k_{6}C_{PGME,2}}{K_{si}}+k3}\right)\\ \left(\frac{k_{2}C_{PGME,2}}{K_{s}}\;+\frac{k_{4b}C_{CEX,2}}{K_{p}}\right)\left(k_{3}+\frac{k_{5}C_{7-ADCA,2}}{K_{n}}\right) \end{array}$$ (18)

where [E] is the concentration of free AEH in the system derived from an enzyme balance (Supplementary Equation S3). Each equation evaluated with the reactor two outlet concentrations of PGME, 7-ADCA, cephalexin, and PG ( $C_{PGME,2},C_{7-ADCA,2},C_{CEX,2},C_{PG,2}$ , respectively) and the concentration of AEH in the reactor, ${\left[E\right]}_{0}$ . The following equations

$$\begin{array}{c} R_{\mathrm{CEX},2}=-R_{7-\mathrm{ADCA},2} \end{array}$$ (19)

$$\begin{array}{c} R_{\mathrm{PG},2}=-R_{\mathrm{PGME},2}-R_{\mathrm{CEX},2} \end{array}$$ (20)

$$\begin{array}{c} C_{\mathrm{CEX},2}=X_{7-\mathrm{ADCA},2}C_{7-\mathrm{ADCA},0} \end{array}$$ (21)

$$\begin{array}{c} C_{\mathrm{PG},2}=X_{\mathrm{PGME},2}C_{\mathrm{PGME},0}-C_{\mathrm{CEX},2} \end{array}$$ (22)

$$\begin{array}{c} X_{7-\mathrm{ADCA},2}=\frac{C_{7-\mathrm{ADCA},0}-C_{7-\mathrm{ADCA},2}}{C_{7-\mathrm{ADCA},0}} \end{array}$$ (23)

$$\begin{array}{c} X_{\mathrm{PGME},2}=\frac{C_{\mathrm{PGME},0}-C_{\mathrm{PGME},2}}{C_{\mathrm{PGME},0}} \end{array}$$ (24)

are required to completely solve the design equation. $C_{7-ADCA,0}$ , $C_{PGME,0}$ , *[E]*_*0*_ *,* $X_{7-ADCA,1}$ *,* and $X_{7-ADCA,2}$ were all specified to solve the system of equations. Finally, residence times of the first and second CSTR ( $\tau_{CSTR,1}$ and $\tau_{CSTR,2}$ , respectively), productivity, and fractional yield were solved using

$$\begin{array}{c} \tau_{\mathrm{CSTR},1}=\frac{X_{7-\mathrm{ADCA},1}C_{7-\mathrm{ADCA},0}}{\left(-R_{7-\mathrm{ADCA},1}\right)} \end{array}$$ (25)

$$\begin{array}{c} \tau_{\mathrm{CSTR},2}=\frac{\left(X_{7-\mathrm{ADCA},2}-X_{7-\mathrm{ADCA},1}\right)C_{7-\mathrm{ADCA},0}}{\left(-R_{7-\mathrm{ADCA},2}\right)} \end{array}$$ (26)

$$\begin{array}{c} \mathrm{Fractional Yield}=\frac{C_{\mathrm{CEX},2}}{C_{\mathrm{PGME},0}-C_{\mathrm{PGME},2}} \end{array}$$ (27)

$$\begin{array}{c} \mathrm{Productivity}\;\left(g/L/\mathrm{hr}\right)=\frac{\left(347.4\frac{g}{\mathrm{mol}}\right)\left(60\frac{\min}{\mathrm{hr}}\right)C_{\mathrm{CEX},2}}{\left(1000\frac{\mathrm{mmol}}{\mathrm{mol}}\right)\left(\tau_{\mathrm{CSTR},1}+\tau_{\mathrm{CSTR},2}\right)} \end{array}$$ (28)

Finally, the CSTR + PFR system was solved using Eqs. 1–9 to describe the CSTR and the PFR was solved using *ode45* for the rate equations with the inlet condition being the outlet concentrations from the CSTR to directly solve for $\tau_{PFR,2}$ , $C_{PGME,2}$ , $C_{7-ADCA,2},$ $C_{CEX,2},$ and $C_{PG,2}$ . $X_{7-ADCA,2}$ and $X_{PGME,2}$ are solved using Eq. 23 and Eq. 24 $\tau_{CSTR,1}$ is calculated from Eq. 10, fractional yield is calculated from Eq. 27, and productivity is calculated as

$$\begin{array}{c} \mathrm{Productivity}\;\left(g/L/\mathrm{hr}\right)=\frac{\left(347.4\frac{g}{\mathrm{mol}}\right)\left(60\frac{\min}{\mathrm{hr}}\right)C_{\mathrm{CEX},2}}{\left(1000\frac{\mathrm{mmol}}{\mathrm{mol}}\right)\left(\tau_{\mathrm{CSTR},1}+\tau_{\mathrm{PFR},2}\right)} \end{array}$$ (29)

## 3 Results

### 3.1 AEH Deactivation

**FIGURE 2:**
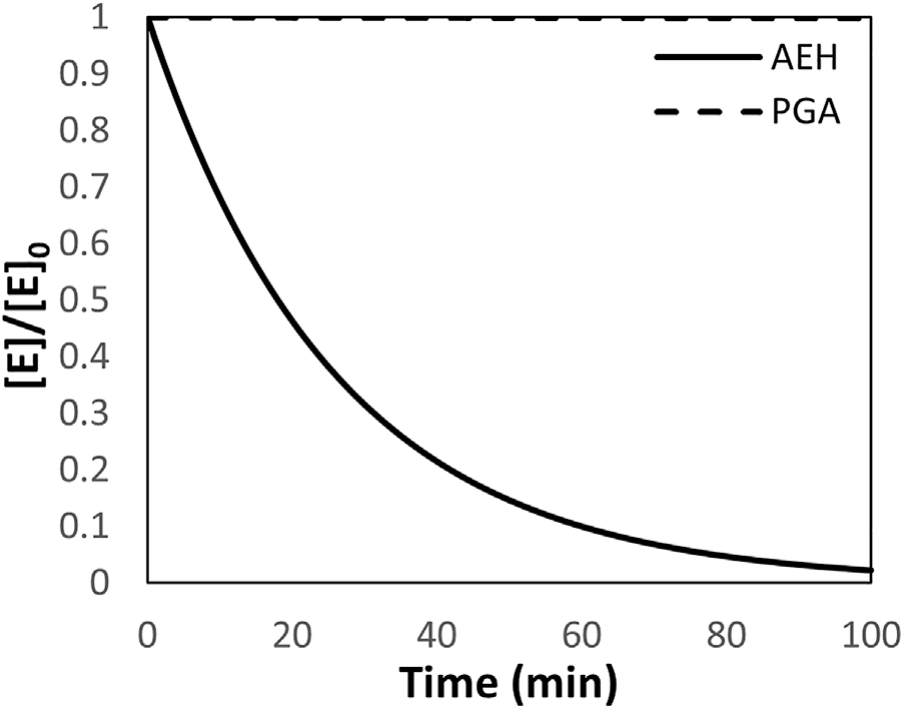
The deactivation of AEH and PGA depicted as the ratio of enzyme at time, t, relative to the initial concentration of enzyme assuming first order deactivation kinetics. The deactivation constant for AEH is 0.0386 min^−1^ while the deactivation constant for PGA is 7E-8 min^−1^ (McDonald et al., 2018). PGA is shown for comparison.

One of the primary assumptions in the design and simulation of a reactor system built around AEH is a constant enzyme concentration. However, AEH deactivates very rapidly at 25°C and pH 7.0 where 50% of activity is lost in around 20 min based on 1^st^ order deactivation kinetics (Figure 2) (Lagerman et al., 2021). For comparison, PGA, a more readily used enzyme for synthesis of *ß*-lactam antibiotics, has <1% deactivation over 100 min under these conditions based on current deactivation data (McDonald et al., 2018). In the following simulations, it is assumed that AEH can be replaced at a rate that compensates for deactivation to assume constant enzyme activity in the reactor.

### 3.2 Single Reactor Simulations

In the following discussion, Levenspiel plots were used to easily visualize the relationship between substrate conversion, reaction rates, and reactor residence times. Levenspiel plot curves are constructed as the ratio of initial substrate concentration divided by the rate of substrate consumption, *C*_7-ADCA,0_ */*(*-R*_7-ADCA_), plotted as a function of conversion, *X*_*7-ADCA*_, and used to determine the residence time, *τ*, of each reactor at a given conversion. As reciprocal rates are used in Levenspiel plots, a negative slope signifies increasing reaction rates with increasing conversion. For a PFR, residence time (Figure 4B, red line) is calculated as the area under the Levenspiel curve for a given conversion (Figure 4A, red line). For a CSTR, residence time (Figure 4B, black line) is calculated as the product of the conversion and corresponding *C*_7-ADCA,0_ */*(*-R*_7-ADCA_), or the area of the rectangle under the Levenspiel curve (Figure 4A, black line).

#### 3.2.1 Single CSTR

A single CSTR is perhaps the simplest operation of an enzymatic reactor and has been simulated and operated for PGA previously (McDonald et al., 2019a; McDonald et al., 2019b). Here, simulations involving AEH were performed by varying reactor residence times and inlet PGME and 7-ADCA concentrations from 25–1,000 mM and 25–500 mM respectively to study how conversion, fractional yield, and productivity are affected by easily tunable operating conditions. Focus was given to maximizing 7-ADCA conversion as the cost of 7-ADCA is much greater than the cost of PGME.

**FIGURE 3:**
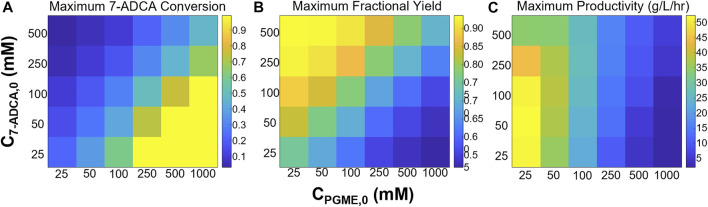
Maximum **(A)** 7-ADCA conversion, **(B)** fractional yield of cephalexin from PGME, and **(C)** productivity in g/L/hr is shown for different combinations of inlet substrate concentrations for a single CSTR. PGME concentrations range from 25–1,000 mM while 7-ADCA concentrations range from 25–500 mM.

Figure 3 shows the maximum 7-ADCA conversions (Figure 3A), maximum fractional yields (Figure 3B), and maximum productivities (Figure 3C) obtainable using a single CSTR for various combinations of inlet PGME and 7-ADCA concentrations. The highest maximum 7-ADCA conversions were found to occur at high inlet concentrations of PGME relative to 7-ADCA. When PGME is in excess, most of the 7-ADCA can be converted to cephalexin despite some PGME being converted to byproduct, PG. Greater than 99% of 7-ADCA can be converted to cephalexin when PGME is supplied at a 10:1 ratio (or greater) of PGME:7-ADCA. When PGME and 7-ADCA are supplied at equal concentrations to the inlet of the reactor, 7-ADCA conversion does not exceed 40% due to low fractional yield of PGME to cephalexin.

Maximum fractional yields (that is, the ratio of moles of cephalexin to moles of PG plus moles of cephalexin) can exceed 0.9 when 7-ADCA is supplied in large excess relative to PGME in contrast to excess PGME giving the highest maximum 7-ADCA conversion; however, it should be noted that the maximum fractional yield for a given combination of inlet concentrations can only be obtained at low conversion (<1%) when 7-ADCA remains at high concentration in the reactor. As more 7-ADCA is converted, selectivity toward cephalexin relative to PG production is decreased resulting in lower fractional yield at higher 7-ADCA conversion. In other words, to obtain the maximum 7-ADCA conversion for a given set of substrate inlet concentrations, fractional yield must be sacrificed and vice versa.

The largest maximum productivities (Figure 3C) were found to occur at low concentrations of both PGME and 7-ADCA with the highest cephalexin productivities occurring in a CSTR with only 25 mM PGME. As the concentration of PGME is increased, productivity drops off substantially as PGME inhibition increases. In other words, to produce the same amount of cephalexin with higher inlet concentrations of PGME, a much longer residence time is required due to the slower rates of reaction caused by substrate inhibition which results in lower productivities. In addition, at low PGME concentrations, 7-ADCA concentration has little effect on productivity unless in large excess (>250 mM). However, at high PGME concentration (>250 mM), increases in inlet 7-ADCA concentration leads to slightly higher productivities due to the higher production of cephalexin that occurs when higher 7-ADCA is supplied.

**FIGURE 4:**
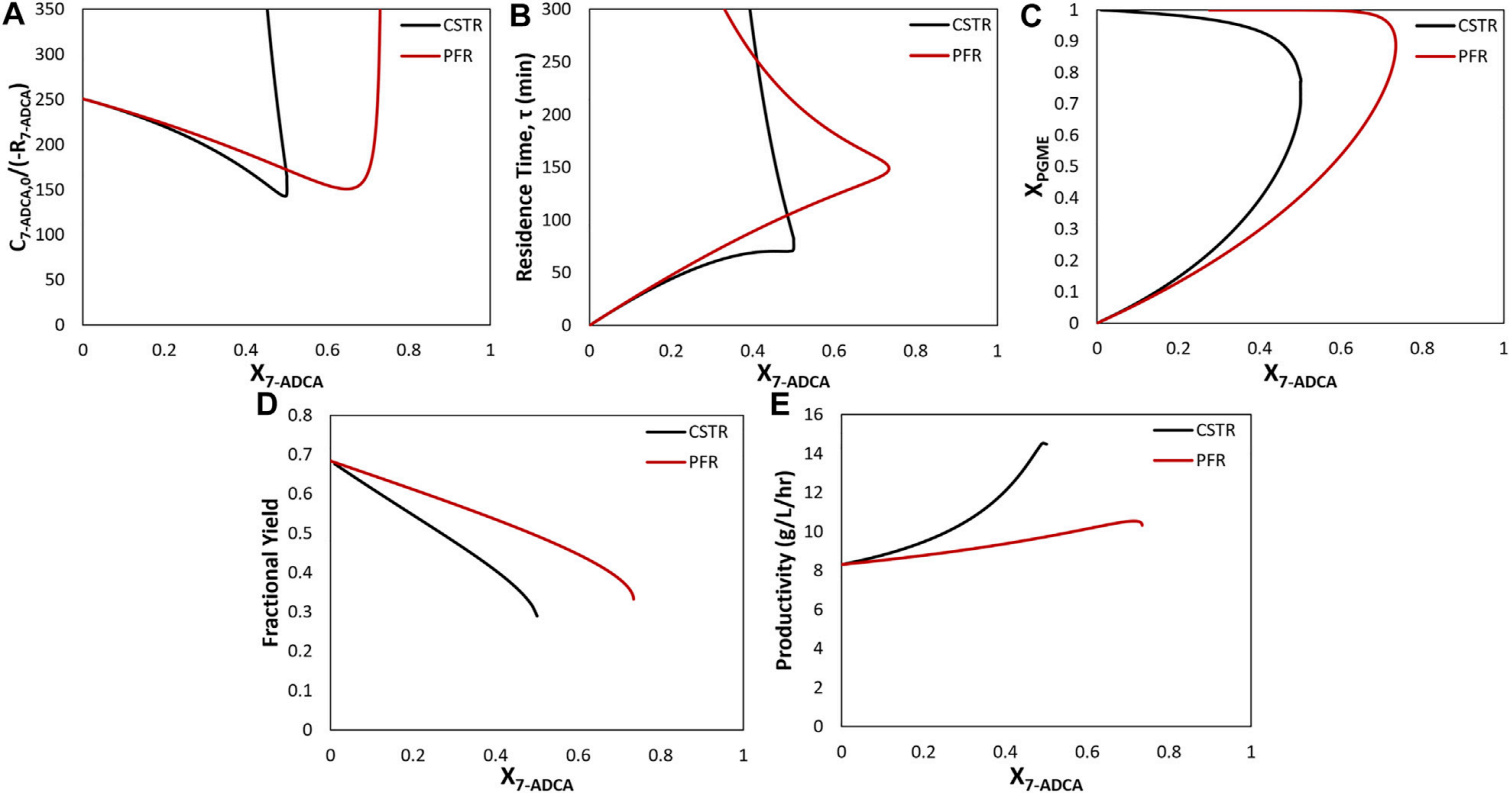
Comparison of a CSTR (black) and PFR (red) for inlet conditions of 250 mM PGME and 100 mM 7-ADCA **(A)** Levenspiel plots showing the inverse 7-ADCA reaction rate as a function of 7-ADCA conversion (*X*_*7-ADCA*_) **(B)** Residence times (τ) as a function of 7-ADCA conversion (*X*_*7-ADCA*_) derived from the Levenspiel plots. Residence time is calculated as the area under the curve for a PFR at a given *X*_*7-ADCA*_. For the CSTR, residence time is calculated as the product of *X*_*7-ADCA*_ and the corresponding *C*_*7-ADCA*_ */*(*-R*_*7-ADCA*_) or the area of the rectangle under the curve at a given *X*_*7-ADCA*_ **(C)** PGME conversions (*X*_*PGME*_) corresponding to each *X*_*7-ADCA*_ in panel **(A)**. **(D)** Fractional yields obtained for *X*_*7-ADCA*_ in panel **(A)**. **(E)** Productivities obtained from Eq. 12 for the CSTR and Eq. 15 for the PFR at all *X*_*7-ADCA*_ values.

The maximum productivity for a given set of inlet concentrations is obtained at the maximum conversion for all combinations of substrate concentrations. While data for all substrate concentrations is not shown, a representative example can be found in Figure 4 (black curves). At high 7-ADCA conversion and therefore high PGME conversion (Figure 4C), the cephalexin synthesis rate achieves a maximum value (minimum y-value) as shown for the example Levenspiel plot for a single CSTR operating with an inlet concentration of 100 mM 7-ADCA and 250 mM PGME (Figure 4A, black curve). The corresponding productivity is also at a maximum at this conversion (Figure 4E, black curve). As PGME is consumed (i.e. higher *X*_*PGME*_), substrate inhibition decreases and the rate of reaction increases, so a CSTR configured with a larger residence time for higher *X*_*7-ADCA*_ operates at a higher rate of reaction and therefore higher productivity than a CSTR operating at low *X*_*7-ADCA*_.

#### 3.2.2 Single PFR

For many of the following comparisons of reactor configuration, an example system with inlet concentrations of 250 mM PGME and 100 mM 7-ADCA at a constant reactor enzyme concentration of 200 nM is used to demonstrate representative trends across all inlet substrate concentration. Figure 4 shows simulation results for a single CSTR and single PFR using the example inlet concentrations. Based on the Levenspiel plot for 250 mM PGME, 100 mM 7-ADCA (Figure 4A), a CSTR operates at a higher reaction rate than a PFR at identical 7-ADCA conversions (*X*_*7-ADCA*_). Because of the higher reaction rates, a CSTR provides a more efficient synthesis than a PFR in terms of lower residence time (Figure 4B) and higher productivity (Figure 4E) for the same *X*_*7-ADCA*_. A PFR, however, allows for higher conversion of 7-ADCA relative to a CSTR (Figure 4A). For this example, a CSTR can only be used to convert 50% of 7-ADCA whereas a PFR can drive conversion up to 73%.

A PFR will have a higher concentration of 7-ADCA throughout the reactor compared to a CSTR because a CSTR operates at the exit conditions of the reactor whereas a PFR is used to gradually consume substrate. For a PFR, this higher 7-ADCA concentration leads to more efficient PGME consumption relative to 7-ADCA consumption (Figure 4C) which in turn leads to much higher fractional yield toward cephalexin when compared to a CSTR (Figure 4D). The reaction rate for a PFR, however, is always lower than that of a CSTR, so a higher residence time is required to reach high conversions (Figure 4B). Productivity is much lower due to the high residence times required to reach a given conversion (Figure 4E). A single CSTR can reach nearly 15 g/L/hr at 50% 7-ADCA conversion whereas a PFR operating at 50% 7-ADCA conversion can only reach about 10 g/L/hr.

### 3.3 Multiple Reactor Simulations

Reactors in series are often used to reach higher substrate conversion than is possible in a single reactor, and CSTRs and PFRs can be used interchangeably in series. Design of reactors in series is often focused on minimization of total reactor volume or total reactor residence time, and residence time is minimized by adjusting conversion in each reactor to achieve the same total conversion. While focus is often given to improving conversion, for AEH-catalyzed cephalexin synthesis, consideration must also be given to tradeoffs in fractional yield and productivity in addition to substrate conversion.

#### 3.3.1 Two CSTRs in Series

**FIGURE 5:**
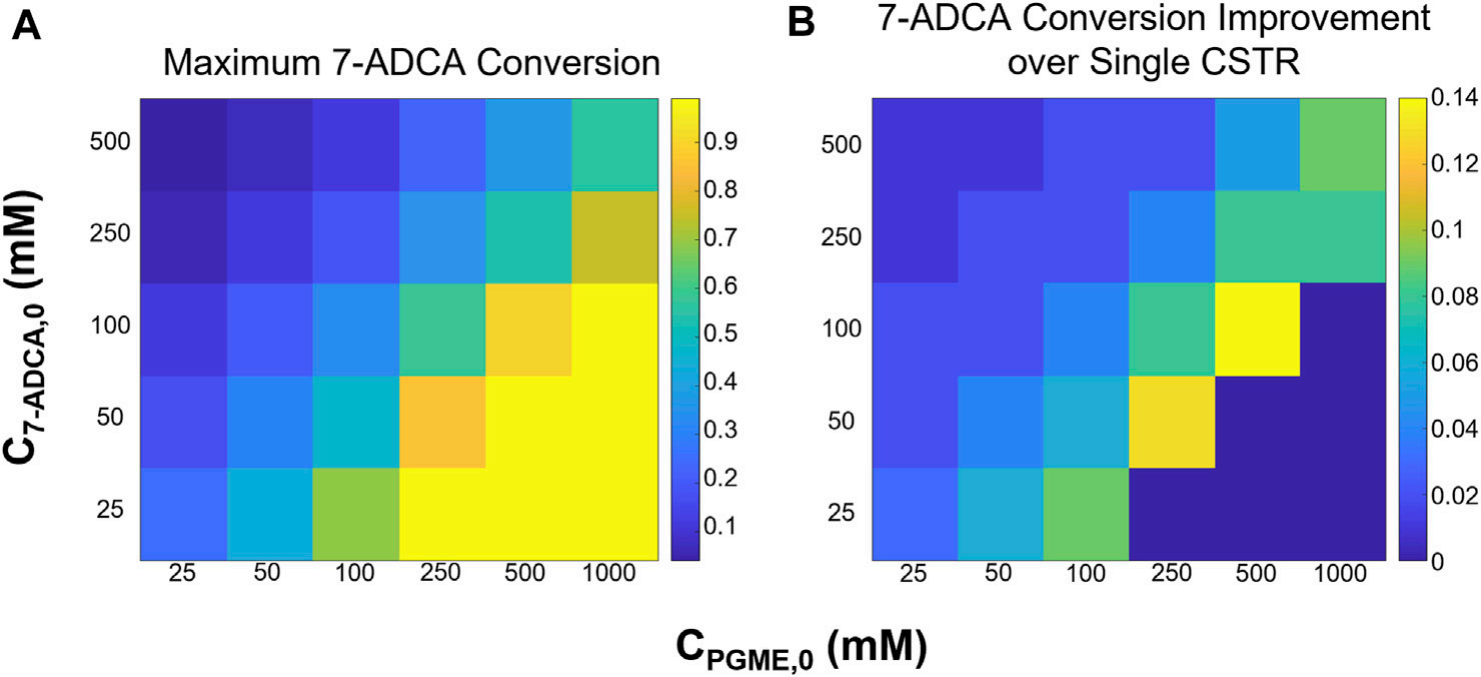
**(A)** Maximum 7-ADCA conversion and **(B)** improvement in maximum 7-ADCA conversion when compared to a single CSTR is shown for a two CSTR system for several combinations of inlet substrate concentrations. PGME concentrations range from 25–1,000 mM while 7-ADCA concentrations range from 25–500 mM.

Two CSTRs in series were simulated while varying 7-ADCA conversion in the first CSTR (*X*_*7-ADCA,1*_) and the total 7-ADCA exiting the second CSTR (*X*_*7-ADCA,2*_). Figure 5 shows the maximum attainable 7-ADCA conversion (Figure 5A) and improvement in maximum conversion (Figure 5B) defined as the difference in total conversion for a CSTR + CSTR system relative to a single CSTR. In general, addition of a second CSTR allows for higher possible 7-ADCA conversion than a single CSTR for all inlet substrate concentrations. At intermediate concentrations of both 7-ADCA and PGME, possible 7-ADCA conversion is increased by up to 14% points. At low 7-ADCA inlet concentrations and high PGME concentrations, 7-ADCA conversions already reach >99%, so improvements are limited, and a second reactor adds no benefit to 7-ADCA conversion. For the previous example of 250 mM PGME and 100 mM 7-ADCA, 7-ADCA conversion is increased from 50 to 58%.

**FIGURE 6:**
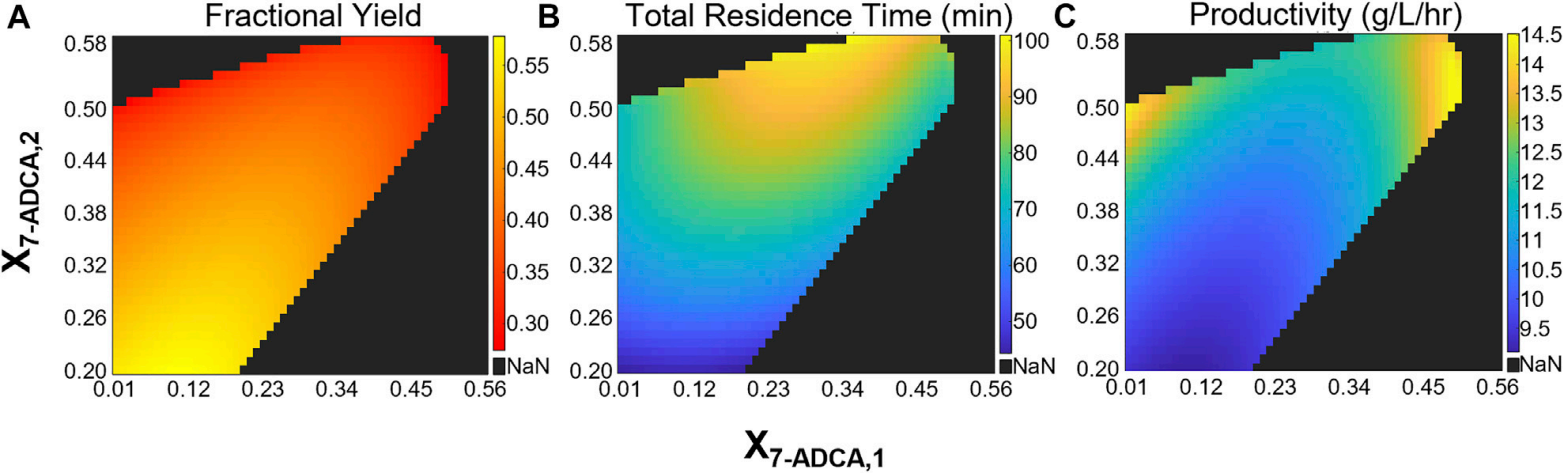
Heatmaps showing the **(A)** fractional yield **(B)** productivity and **(C)** total residence time for a CSTR + CSTR system at various combinations of 7-ADCA conversions in each reactor. The *x*-axis shows the total conversion reached in the first CSTR and the *y*-axis shows the total 7-ADCA conversion exiting reactor two assuming an inlet 7-ADCA concentration of 100 mM and inlet PGME concentration of 250 mM. The AEH concentration in both reactors is constant at 200 nM.

Two CSTRs in series allow for an additional degree of freedom as each reactor can be sized separately while achieving the same total 7-ADCA conversion. To study how the configuration of the first CSTR impacts productivity, fractional yield, and total residence time, a two-CSTR system was simulated at several total 7-ADCA conversions (*X*_*7-ADCA,2*_) while varying conversion achieved in the first CSTR (*X*_*7-ADCA,1*_) using the example inlet conditions of 250 mM PGME and 100 mM 7-ADCA and constant enzyme concentration of 200 nM in each reactor. Figure 6 shows the fractional yield (Figure 6A), total residence time (Figure 6B), and productivity (Figure 6C) obtained for several combinations of CSTR configurations. As established for a single CSTR, high total 7-ADCA conversion (*X*_*7-ADCA,2*_) limits fractional yield and no combination of two CSTRs operating at a high *X*_*7-ADCA,2*_ (Figure 6A) improves fractional yield significantly over a single CSTR (Figure 4B, black curve). For example, when total 7-ADCA conversion is 0.58, varying *X*_*7-ADCA,*1_ does not improve fractional yield above 0.3. Significant improvement in fractional yield can be obtained at lower total 7-ADCA conversions (*X*_*7-ADCA,2*_ ≤ 0.50) when 50% of the total conversion is achieved in the first CSTR (i.e. *X*_*7-ADCA,1*_ ≈ 0.5*X*_*7-ADCA,2*_) (Figure 6A). Such a configuration of CSTRs, however, impacts productivity and residence time significantly. With two similarly sized CSTRs achieving similar conversion (*X*_*7-ADCA,1*_ ≈ 0.5*X*_*7-ADCA,2*_), the total residence time is maximized (Figure 6B), and productivity is minimized (Figure 6C). For example, when *X*_*7-ADCA,2*_ = 0.50, the total residence time is approximately 75 min and productivity is 14.5 g/L/hr when *X*_*7-ADCA,1*_ < 0.05 or when *X*_*7-ADCA,1*_ > 0.45 compared to a residence time of 92 min and productivity of 11 g/L/hr when *X*_*7-ADCA,1*_ = 0.25. In other words, productivity is maximized when one CSTR is much smaller than the other to minimize total residence time. Overall, a second small CSTR should only be used to drive conversion farther than a single CSTR is capable.

#### 3.3.2 Comparison of Reactors in Series to Single CSTR and Single PFR

**FIGURE 7:**
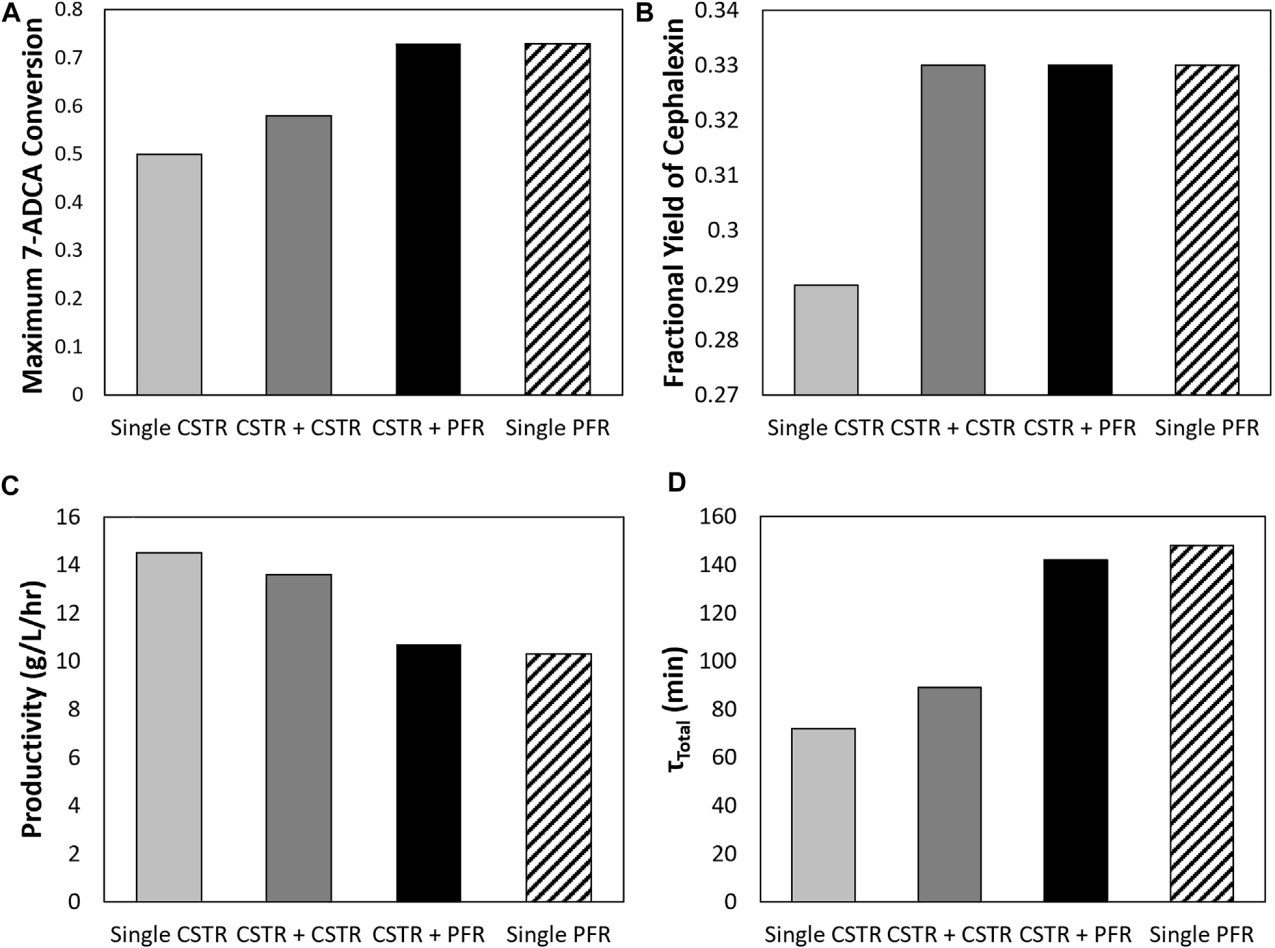
A comparison is shown of the **(A)** maximum 7-ADCA conversion as well as the **(B)** fractional yield at the maximum 7-ADCA conversion, **(C)** productivity at maximum 7-ADCA conversion, and **(D)** total residence time at maximum 7-ADCA conversion for an inlet reactor concentration of 250 mM PGME and 100 mM 7-ADCA and AEH concentration of 200 nM is shown for the single CSTR, single PFR, CSTR plus CSTR, and CSTR plus PFR systems.

Figure 7 shows how different reactor configurations impact maximum 7-ADCA conversion (Figure 7A) and the associated fractional yields (Figure 7B) and productivities (Figure 7C) at the maximum conversion for inlet substrate concentrations of 250 mM PGME and 100 mM 7-ADCA at a constant AEH concentration of 200 nM in all reactors. As mentioned previously, two CSTRs can drive 7-ADCA conversions higher to 58% when compared to a single CSTR conversion of 50%. While driving 7-ADCA conversion higher typically has negative impact on fractional yield due to the lower concentration of 7-ADCA in the reactor, the two CSTR configuration shows slightly improved fractional yield at 58% 7-ADCA conversion than a single CSTR at 50% conversion. The increase in conversion, however, does have a slight negative impact on productivity, reducing cephalexin productivity from 14.5 g/L/hr to 13.6 g/L/hr. The addition of a CSTR prior to a PFR provides no advantage to fractional yield or 7-ADCA conversion than a PFR alone; however, the CSTR helps reduce total residence time and thus provides a higher overall productivity when compared to a PFR operating at the same conversion.

### 3.4 Effects of Enzyme Concentration on Reactor Design

**FIGURE 8:**
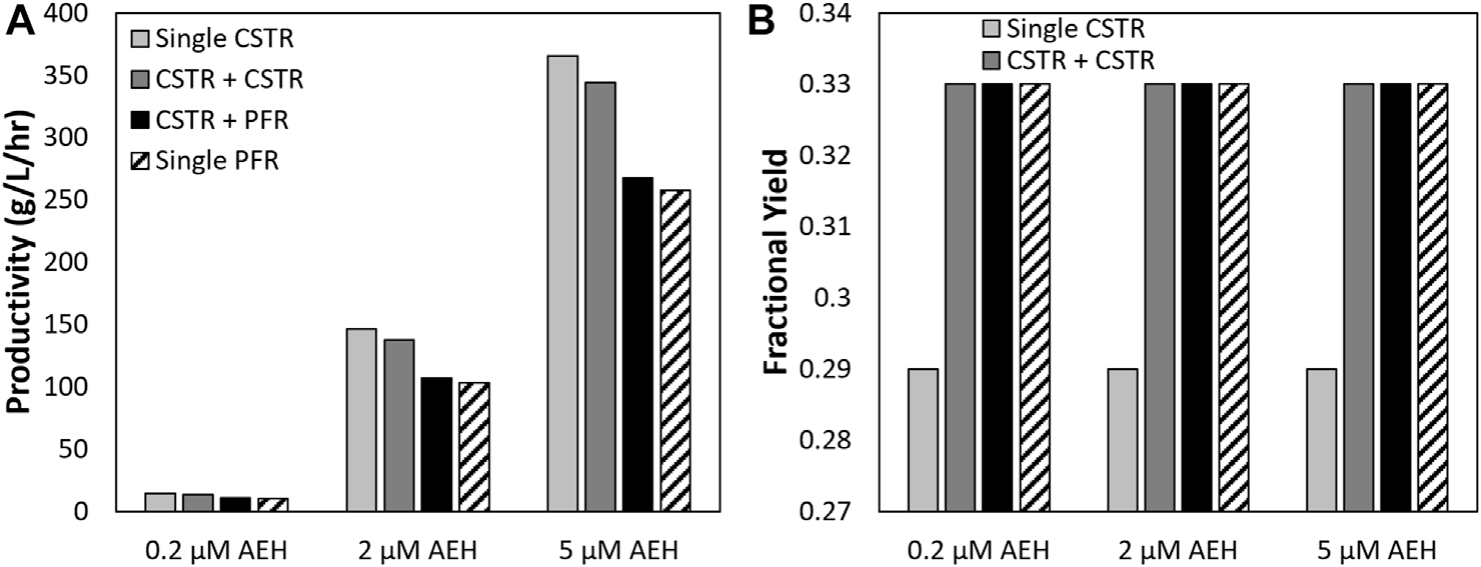
**(A)** productivities and **(B)** fractional yields obtained for a single CSTR, single PFR, and CSTR + PFR are shown at varying concentrations of AEH at the maximum 7-ADCA conversion for each respective reactor configuration assuming 250 mM PGME and 100 mM 7-ADCA inlet concentrations.

AEH concentration was varied using the 250 mM PGME, 100 mM 7-ADCA example to study how the concentration of enzyme affects productivity, 7-ADCA conversion, and fractional yield. Figure 8 shows the impact of AEH concentration on productivity. Productivity is proportional to the concentration of enzyme supplied to the reactor as more enzyme increases the speed of the reaction and thus requires a much smaller residence time to convert the same amount of reactant as a smaller concentration of enzyme would require. This in turn increases cephalexin productivity and has no impact on fractional yield (Figure 8B) as the rate of PG and cephalexin production scale equally with enzyme concentration. This data shows that fractional yield and conversion can be set by the reactor conditions (residence time, inlet concentrations, reactor configurations, etc.) and productivity can be scaled accordingly with the concentration of enzyme to match production requirements.

### 3.5 Simulation of a Single CSTR With an Improved AEH

**FIGURE 9:**
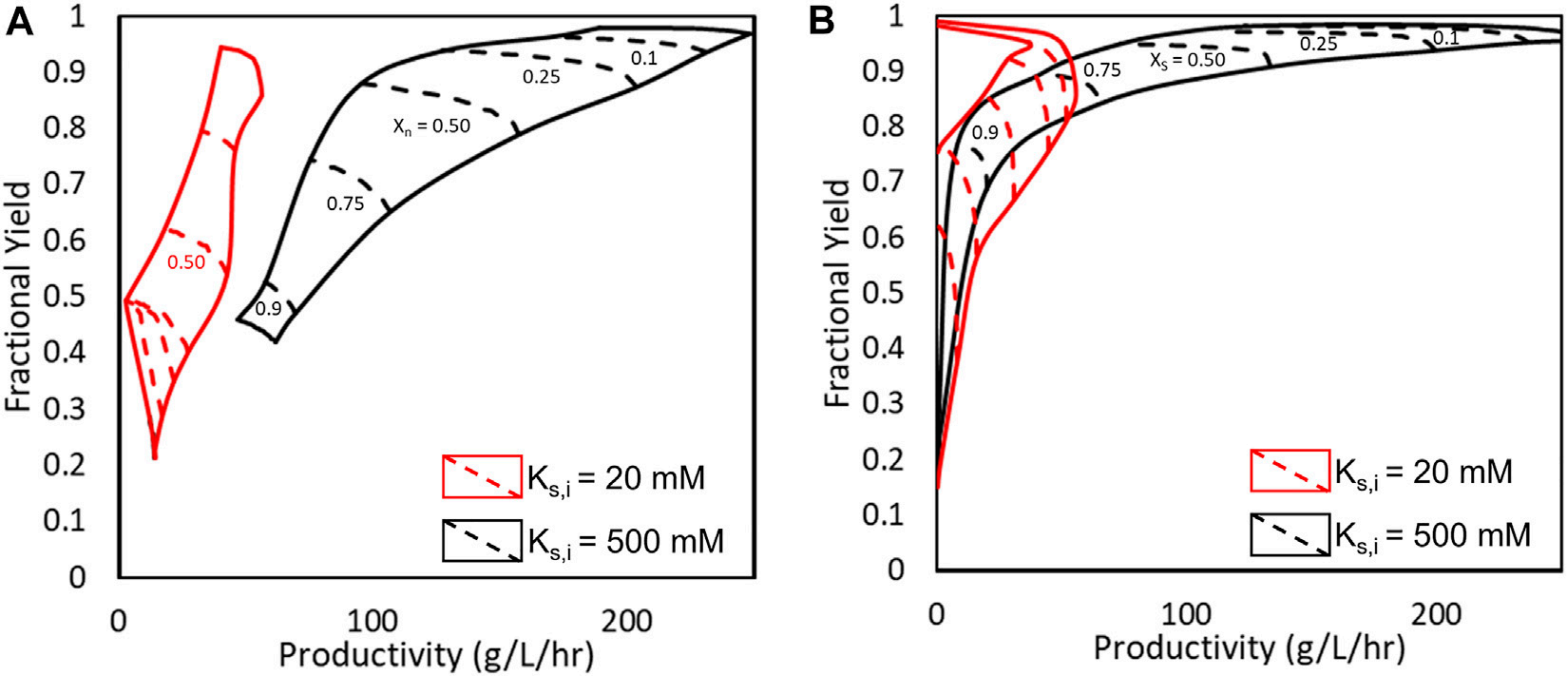
Pareto optimal fronts for fractional yield and productivity under **(A)** varying 7-ADCA conversion (*X*_*7-ADCA*_) and **(B)** varying PGME conversion. The red curves show the obtainable fractional yields, productivities, and conversions for the current AEH (*K*_*S,I*_ = 20 mM) while the black curves represent an engineered AEH with minimized substrate inhibition (*K*_*S,I*_ = 500 mM).

Finally, to demonstrate the impacts of substrate inhibition on AEH catalyzed cephalexin synthesis, an engineered AEH was simulated assuming substrate inhibition could be reduced through protein engineering. This was simulated by increasing *K*_*S,I*_ to 500 mM while keeping all other model parameters constant. In Figure 9, Pareto plots comparing 7-ADCA conversion (Figure 9A) and PGME conversion (Figure 9B) to both fractional yield and productivity. The Pareto curves show either the maximum fractional yields or productivities that can be obtained without sacrificing the other parameter at a given conversion by changing the reactor conditions and still obtaining a given conversion. The red curves show the Pareto curves for a single CSTR simulated with wildtype AEH from *Xanthomonas campestris pv. campestris.* (Lagerman et al., 2021), and the black curves show the hypothetical improved AEH with reduced substrate inhibition. All Pareto plots are simulated with 200 nM AEH.

With the wildtype AEH, high conversion of 7-ADCA cannot be reached without sacrificing both fractional yield and productivity. Past 50% 7-ADCA conversion, equal amounts of both byproduct PG and cephalexin are produced rendering an overall inefficient process with just a single CSTR as previously discussed. With an improved AEH, a single CSTR can be designed for 75% conversion of 7-ADCA while still reaching fractional yields up to 75% and productivities between 80–110 g/L/hr. The single CSTR operated with an improved AEH can also reach much higher conversions of PGME while maintaining high fractional yield, which is not possible with the current AEH.

## 4 Discussion and Conclusion

The design of an efficient reactor network for the synthesis of cephalexin catalyzed by AEH is a difficult task due to rapid deactivation of AEH (Figure 2) and strong substrate inhibition by PGME. While addition of AEH over time into the reactor is a possible solution for constant enzyme concentration, such a process would be relatively expensive and, overall, an infeasible solution. Instead, improvements to the stability of AEH can be achieved through protein engineering and should be pursued to further enable AEH catalyzed synthesis of cephalexin.

Substrate inhibition should also be a focus for AEH engineering efforts. Based on the current kinetic model, the inhibition constant for AEH toward PGME (*K*_*S,I*_) is 20 mM and reactor configurations require PGME well above 100 mM for selective and efficient production of cephalexin (Figure 3). Further understanding of the underlying mechanism for PGME inhibition and modifications to alleviate this inhibition are necessary to further pursue AEH for synthesis of cephalexin. If substrate inhibition can be reduced through protein engineering, a single CSTR could be operated to obtain high fractional yield, substrate conversion, and productivity (Figure 8). Other reactor configurations may further optimize use of an improved AEH; however, this is currently outside the scope of this work.

Careful design of an AEH-catalyzed reactor network can help alleviate the effects of substrate inhibition with the current AEH; however, tradeoffs between high substrate conversion and high fractional yield still exist. While maintaining a high inlet ratio of 7-ADCA to PGME improves selectivity toward cephalexin, doing so reduces maximum 7-ADCA conversion (Figures 3, 4, 6) and leads to a large amount of wasted substrate. As 7-ADCA is the more expensive reactant, conversion of 7-ADCA should be maximized without sacrifice of selectivity toward cephalexin which is currently not attainable given the current substrate inhibition.

Based on these simulations, no single optimum design can be found that maximizes productivity, fractional yield, and 7-ADCA conversion (Figure 3). With a single CSTR, the highest productivity is achieved at low concentrations of both 7-ADCA and PGME as substrate inhibition is not as prevalent (Figure 3C). High fractional yield occurs at high concentrations of 7-ADCA and low concentrations of PGME to shift production away from PG and toward cephalexin (Figure 3B); however, this comes at a cost to 7-ADCA conversion as PGME becomes the limiting substrate. Finally, high 7-ADCA conversion is attainable with low 7-ADCA and high PGME (Figure 3A), but much more byproduct PG is produced which lowers fractional yield.

A single PFR allows for higher conversion and fractional yield over a single CSTR, but total residence time and productivity suffer (Figure 4). Productivity can be improved through an increase in AEH supplied to the reactor (Figure 7) but operating at high 7-ADCA conversion still reduces fractional yields to inefficient values as the production of PG increases drastically as 7-ADCA is consumed (Figure 4D). Multi-reactor configurations allow for increases in 7-ADCA conversion relative to a single CSTR but cannot achieve higher 7-ADCA conversions than can be obtained with a single PFR. Higher fractional yields are also attainable relative to a single CSTR (Figure 5, Figures 6A, B); however, this conversion increase comes at the cost of productivity (Figure 6C) and increased residence time (Figure 6D). Ultimately, a single PFR provides the highest fractional yield at the highest 7-ADCA conversion and should be considered for AEH catalyzed synthesis of cephalexin.

## Data Availability

Publicly available datasets were analyzed in this study. This data can be found here: doi.org/10.1016/j.cej.2021.131816.
