# Supplementary material for: Reactor Design and Optimization of α-Amino Ester Hydrolase- Catalyzed Synthesis of Cephalexin
Source: Front Bioeng Biotechnol. 2022 Mar 2;10:826357. doi: 10.3389/fbioe.2022.826357 (PMC8924468; doi:10.3389/fbioe.2022.826357)
Supplement: Supplementary file 1 [file DataSheet1.docx]

**Supplementary Information**

The general rate equations used to define AEH-catalyzed synthesis of cephalexin are, with equations 2 and 3 from the main text

$$\begin{aligned} R_{CEX}=\frac{k_{2}\left[ E \right]C_{PGME}}{K_{s}}-\left( \frac{\left[ E \right]}{\frac{k_{4}C_{7\text{-}ADCA}}{K_{n}}+\frac{k_{5}C_{7\text{-}ADCA}}{K_{n}}+\frac{k_{6}C_{PGME}}{K_{si}}+k3} \right)\left( \frac{k_{2}C_{PGME}}{K_{s}} +\frac{k_{4b}C_{CEX}}{K_{p}} \right)\left( k_{3}+\frac{k_{5}C_{7\text{-}ADCA}}{K_{n}} \right)\#\left( S1 \right) \end{aligned}$$

$$\begin{aligned} R_{PG}=\left( \frac{\left[ E \right]}{\frac{k_{4}C_{7\text{-}ADCA}}{K_{n}}+\frac{k_{5}C_{7\text{-}ADCA}}{K_{n}}+\frac{k_{6}C_{PGME}}{K_{si}}+k3} \right)\left( \frac{k_{2}C_{PGME}}{K_{s}} +\frac{k_{4b}C_{CEX}}{K_{p}} \right)\left( k_{3}+\frac{k_{5}C_{7\text{-}ADCA}}{K_{n}} +\frac{k_{6}C_{PGME}}{K_{si}} \right)\#\left( S2 \right) \end{aligned}$$

$$\begin{aligned} \left[ E \right]=\frac{\left[ E \right]_{0}}{1+\frac{{C_{PGME}}^{2}}{K_{s}K_{si}}+\frac{C_{7\text{-}ADCA}}{K_{n}}+ \left( \frac{k_{2}C_{PGME}}{K_{s}\left( \frac{k_{4}C_{7\text{-}ADCA}}{K_{n}}+\frac{k_{5}C_{7\text{-}ADCA}}{K_{n}}+\frac{k_{6}C_{PGME}}{K_{si}}+k3 \right)} \right)\left( 1+\frac{C_{7\text{-}ADCA}}{K_{n}}+\frac{C_{PGME}}{K_{si}} \right)+\frac{C_{CEX}}{K_{p}}+\frac{C_{PG}}{K_{p2i}}}\#\left( S3 \right) \end{aligned}$$

$R_{CEX}$ is the molar rate of cephalexin reacted, $R_{PG}$is the molar rate of PG reacted, and $\left[ E \right]$ is the concentration of free AEH in the system derived from an enzyme balance. It should be noted that all rate equations account for all reactions in the network shown in Scheme 1. For example, $R_{CEX}$ accounts for both cephalexin production and consumption and $R_{7\text{-}ADCA}$ accounts for both 7-ADCA production and consumption. The parameters for the reactor model can be found below in Table S1.

Table S1: Model Parameters for the Reaction Network in Scheme 1

| Parameter | Units | Value |
| --- | --- | --- |
| *K_S_* | mM | 14 |
| *k_2_* | s^-1^ | 432 |
| *K_N_* | mM | 290 |
| *k_3_* | s^-1^ | 417 |
| *k_4_* | s^-1^ | 73600 |
| *k_5_* | s^-1^ | 491 |
| *k_6_* | s^-1^ | 1660 |
| *K_Si_* | mM | 20 |
| *K_p_* | mM | 39 |
| *k_-4_* | s^-1^ | 9126 |
| [*E*]*_0_* | mM | 1.98E-04 |
| *k_d_* | s^-1^ | 6.43E-04 |
| *K_PG_* | mM | 12 |


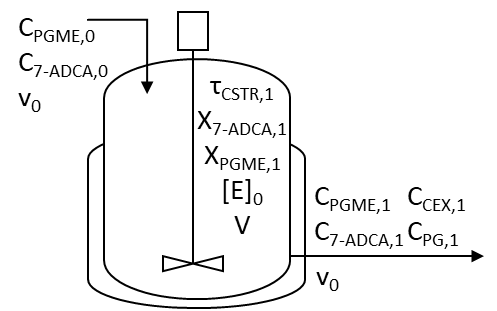


Figure S1: Single CSTR and variables involved in solving the system.

The single CSTR (Figure S1) is solved through a mass balance around the substrates fed to the system. For PGME,

$$\begin{aligned} F_{PGME,0}-F_{PGME,1}+\int^{V} R_{PGME}dV=\frac{dN_{PGME}}{dt}\#\left( S6 \right) \end{aligned}$$

where $F_{PGME,0}$ is the rate of PGME moles added to the CSTR in moles/time, $F_{PGME,1}$is the rate of PGME moles exiting the CSTR in moles/time, $\int^{V} R_{PGME}dV$ is the molar rate of reaction of PGME within the volume, *V,* of a CSTR, where $R_{PGME}$is the total rate of consumption of PGME as defined in Equation S1. $\frac{dN_{PGME}}{dt}$ is the molar rate of accumulation of PGME in the system. Assuming steady state ($\frac{dN_{PGME}}{dt}=0$) and a perfectly mixed CSTR ($\int^{V} R_{PGME}dV=R_{PGME}V$), Equation S1 simplifies to

$$\begin{aligned} F_{PGME,0}-F_{PGME,1}+R_{PGME}V=0\#\left( S7 \right) \end{aligned}$$

$F_{PGME,0}$ and $F_{PGME,1}$can be written in terms of the concentrations and volumetric flow rates of PGME added and removed from the system such that

$$\begin{aligned} F_{PGME,i}=v_{i}C_{PGME,i}\#\left( S8 \right) \end{aligned}$$

where $v_{i}$ is the volumetric flow rate of stream *i*, and $C_{PGME,i}$ is the concentration of PGME in stream *i.* The residence time of a CSTR, $\tau_{CSTR}$ is defined as the ratio of the volume of the CSTR, *V*, to the volumetric flow rate of the inlet and exit to the CSTR which is assumed to be equal and defined as $v_{0}$ (Equation S9).

$$\begin{aligned} \tau_{CSTR}={V/v}_{0}\#\left( S9 \right) \end{aligned}$$

Substituting Equations S8 and S9 into Equation S7 and rearranging yields

$$\begin{aligned} \tau_{CSTR}=\frac{C_{PGME,0}-C_{PGME,1}}{-R_{PGME}}\#\left( S10 \right) \end{aligned}$$

The conversion of PGME can be defined as

$$\begin{aligned} X_{PGME}=\frac{moles of PGME reacted}{moles of PGME fed}=\frac{C_{PGME,0}-C_{PGME,1}}{C_{PGME,0}}\#\left( S11 \right) \end{aligned}$$

Solving for $C_{PGME,1}$ in Equation S11 and substituting into Equation S10, $\tau_{CSTR}$ can be defined as

$$\begin{aligned} \tau_{CSTR}=\frac{C_{PGME,0}X_{PGME}}{-R_{PGME}}\#\left( S12 \right) \end{aligned}$$

An identical process can be taken to obtain $\tau_{CSTR}$ in terms of 7-ADCA to obtain

$$\begin{aligned} X_{7\text{-}ADCA}=\frac{moles of 7\text{-}ADCA reacted}{moles of 7\text{-}ADCA fed}=\frac{C_{7\text{-}ADCA,0}-C_{7\text{-}ADCA,1}}{C_{7\text{-}ADCA,0}}\#\left( S13 \right) \end{aligned}$$

$$\begin{aligned} \tau_{CSTR}=\frac{C_{7\text{-}ADCA,0}X_{7\text{-}ADCA}}{-R_{7\text{-}ADCA}}\#\left( S14 \right) \end{aligned}$$

where $X_{7\text{-}ADCA}$ is the conversion of 7-ADCA, $R_{7\text{-}ADCA}$ is the reaction rate of 7-ADCA, and $C_{7\text{-}ADCA,0}$ is the concentration of 7-ADCA in the inlet to the CSTR. For a given CSTR configuration, a single value for $\tau_{CSTR}$ must satisfy both Equations S12 and S14, so setting these equations equal defines the design equation,

$$\begin{aligned} \frac{C_{7\text{-}ADCA,0}X_{7\text{-}ADCA}}{-R_{7\text{-}ADCA}}=\frac{C_{PGME,0}X_{PGME}}{-R_{PGME}}\#\left( S15 \right) \end{aligned}$$

equivalent to Equation 1 in the main text.

To solve the system, the inlet substrate concentrations $C_{PGME,0}$ and $C_{7-ADCA,0}$, the AEH concentration in the reactor $\left[ E \right]_{0}$, and $X_{7-ADCA}$ are given a value. The outlet concentration of 7-ADCA, $C_{7-ADCA,1}$, is solved using Equation S13 and the mass balance around the β-lactam ring present in only 7-ADCA and cephalexin can be used to solve for the concentration of cephalexin in the outlet $C_{CEX,1}$ such that

$$\begin{aligned} C_{CEX,1}=X_{7\text{-}ADCA,1}C_{7\text{-}ADCA,0}\#\left( S16 \right) \end{aligned}$$

All 7-ADCA that is reacted in the system must be formed as cephalexin according to the mass balance. This is reflected in Equation S16 which is equivalent to Equation 8 in the main text. Similarly, a mass balance can be completed around the phenol ring present in cephalexin, PGME, and the byproduct PG.

$$\begin{aligned} C_{PG,1}=C_{PGME,0}-C_{PGME,1}-C_{CEX,1}= X_{PGME,1}C_{PGME,0}-C_{CEX,1}\#\left( S17 \right) \end{aligned}$$

All PGME reacted goes to either cephalexin or PG, so the outlet concentration of PG must be equal to the initial concentration of PGME minus the remaining PGME and cephalexin exiting the CSTR as shown in Equation S17. This equation can be rewritten in terms of PGME conversion as shown in Equation 9 in the main text. Because a CSTR operates at the outlet conditions of the CSTR (assuming the CSTR is ideal and well mixed), the concentrations of all substrates and products in the outlet stream ($C_{PGME,1},C_{7\text{-}ADCA,1}, C_{PG,1}$, and $C_{CEX,1}$ or their corresponding equations) are used to calculate the rates of reactions by plugging the resulting value for equation S16 for $C_{CEX}$, the resulting value of $C_{7\text{-}ADCA,1}$ from Equation S13 (or Equation 7 from the main text) for $C_{7\text{-}ADCA}$, Equation S17 for $C_{PG}$, and after rearranging for $C_{PGME,1}$, Equation S11 for $C_{PGME}$ in Equations S1 – S4. With given values of $C_{PGME,0}$, $C_{7-ADCA,0}$, $\left[ E \right]_{0}$, and $X_{7\text{-}ADCA}$, Equations S1 and S2 can be plugged into Equation S15 and solved for $X_{PGME}$.

This process can be extended to the two CSTR system by performing a mass balance around both CSTRs such that the inlet to the second CSTR has identical properties to the outlet of the first CSTR which yields equation 16 in the main text.
